# Improvement of Mechanical Properties and Solvent Resistance of Polyurethane Coating by Chemical Grafting of Graphene Oxide

**DOI:** 10.3390/polym15040882

**Published:** 2023-02-10

**Authors:** Guotao Liang, Fengbiao Yao, Yanran Qi, Ruizhi Gong, Rui Li, Baoxuan Liu, Yueying Zhao, Chenglong Lian, Luming Li, Xiaoying Dong, Yongfeng Li

**Affiliations:** 1 State Forestry and Grassland Administration Key Laboratory of Silviculture in Down-Stream Areas of the Yellow River, College of Forestry, Shandong Agricultural University, Tai’an 271018, China; 2 School of Automation, Chongqing University of Posts and Telecommunications, Chongqing 400065, China; 3 Shandong Laucork Development Co., Ltd., Jining 272100, China; 4 Postdoctoral Innovation Practice Base, Shandong Xiaguang Group Co., Ltd., Jining 277600, China; 5 College of Chemistry and Materials Engineering, Zhejiang A&F University, Hangzhou 311300, China

**Keywords:** waterborne polyurethane, mechanical performance, chemical resistance, graphene oxide, chemical graft, wood coatings

## Abstract

Waterborne polyurethane coatings (WPU) are widely used in various types of coatings due to their environmental friendliness, rich gloss, and strong adhesion. However, their inferior mechanical properties and solvent resistance limit their application on the surface of wood products. In this study, graphene oxide (GO) with nanoscale size, large surface area, and abundant functional groups was incorporated into WPU by chemical grafting to improve the dispersion of GO in WPU, resulting in excellent mechanical properties and solvent resistance of WPU coatings. GO with abundant oxygen-containing functional groups and nanoscale size was prepared, and maintained good compatibility with WPU. When the GO concentration was 0.7 wt%, the tensile strength of GO-modified WPU coating film increased by 64.89%, and the abrasion resistance and pendulum hardness increased by 28.19% and 15.87%, respectively. In addition, GO also improved the solvent resistance of WPU coatings. The chemical grafting strategy employed in this study provides a feasible way to improve the dispersion of GO in WPU and provides a useful reference for the modification of waterborne wood coatings.

## 1. Introduction

The adverse effects of traditional solvent-based coatings on the environment and human health have attracted extensive attention. Under the environment of green and sustainable development, the development and utilization of healthy and environmentally friendly waterborne coatings have become a research hotspot. With water as the solvent, waterborne wood coatings are almost no volatile organic compounds and air pollutants, have a wide range of applications, and are safe and reliable in construction, which is favored by practitioners [1,2,3]. Waterborne polyurethane(WPU) coatings are mainly produced by the polyaddition reaction of a polyisocyanate, polyether, or polyol with some chain extenders and hydrophilic agents. It is a new type of polyurethane coating system with water as the solvent or dispersion medium [4,5,6]. WPU has the advantage of good flexibility in the coating film, high and low-temperature resistance, and the advantages of environmental protection. The less or zero release of volatile organic compounds is also in line with the concept of sustainable development, which makes it become a very potential waterborne coating [7].

However, WPU is rich in strong polar groups, which will gradually accumulate heat under cyclic and alternating stress, destroying the cross-linked structure, and thus causing the WPU coating surface to be easily damaged during tension and friction. In addition, the WPU structure contains hydrophilic groups such as carboxyl groups, hydroxyl groups, and ether bonds. The existence of these groups leads to poor solvent resistance of WPU [8,9,10,11]. The disadvantages of weak mechanical properties and solvent resistance limit the application of WPU on the surface of wood products. Scholars have sought methods of crosslinking modification, such as linking linear molecular chain segments in WPU with chemical bonds with strong forces to produce the cross-linking effect. Trimethylolpropane (TMP), triethanolamine, glycerol, and green castor oil (CO) were used as crosslinking agents to improve the mechanical properties and solvent resistance of WPU coatings, respectively [12,13,14,15]. Both acrylate resin (PA) and epoxy resin (EA) have the advantages of high strength and good solvent resistance. Combining them with WPU through physical blending or chemical grafting is of great help to improve the mechanical properties and solvent resistance of polyurethane [16,17,18]. Organic silicon polymers have low surface energy, high and low-temperature resistance, climate aging resistance, and physiological inertia, while organic fluorine polymers have excellent hydrophobic, and oleophobic surface properties and chemical stability. By introducing them into the main chain of WPU, the solvent resistance of WPU can be improved, and the surface enrichment and low-temperature compliance of WPU can be enhanced, to improve the comprehensive performance of the polymer [19,20,21]. In addition, nanomaterials such as nano-silicon dioxide (SiO_2_), nano-hydroxyapatite (MHAPS), nano-zinc oxide (ZnO), nano-cellulose, nano-chitosan have special properties such as surface effect, quantum size effect, strong interface attraction, and macroscopic quantum tunneling effect. Combining WPU substrates with nanofillers by blending, in situ polymerization, sol-gel method, and intercalation polymerization can endow WPU with excellent mechanical and solvent resistance properties [22,23,24,25]. However, the physical blending of nanomaterials and WPU has the problem of weak compatibility [26], so chemical grafting is a potentially feasible option to improve the compatibility of the two.

Graphene oxide (GO), a type of nanomaterial, is easy to prepare and has the advantages of huge specific surface area, excellent mechanical properties, and good dispersion in water [27,28]. As a cross-linking agent, it can be covalently bonded with organic substances. For example, carboxyl groups can undergo an esterification reaction with organic substances containing hydroxyl groups, and can also undergo an amidation reaction with organic molecules containing amino groups. Moreover, hydrogen bonds, π–π bond interaction, intermolecular forces, and other non-covalent bond bonding can also occur between GO and organisms [29,30,31]. Introducing GO into WPU can increase the force between organic molecules, achieving the purpose of improving mechanical properties. GO contains a large number of oxygen-containing functional groups, so it has more reactive sites, which is helpful to improve the dispersion and compatibility of GO and WPU. In addition, the lamellar structure of GO can effectively prolong the penetration path of solvent molecules, thus playing a certain barrier role, and further helping the solvent resistance of the coating film to a certain extent [32].

**Figure 1:**
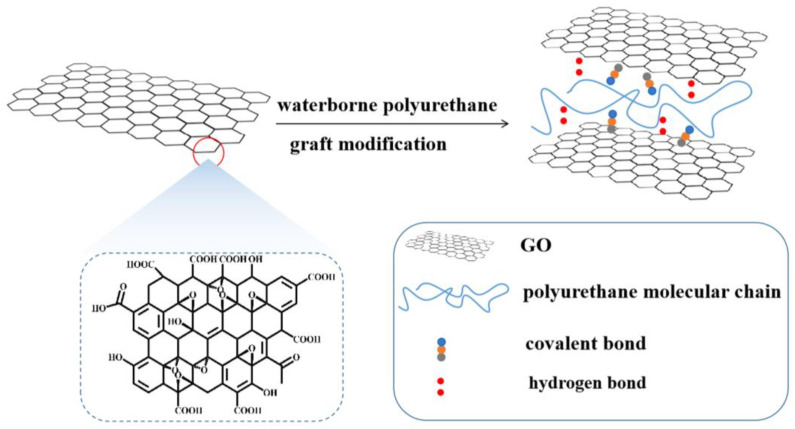
GO modified WPU schematic diagram.

The physical blending [33] of GO or modified GO with dopamine and with WPU [34] can improve the mechanical properties and barrier properties of the coating [35,36]. However, physical blending will result in poor compatibility and dispersion of GO and WPU, and the modification ability of GO cannot be effectively exerted, which will easily cause stress concentration and get the opposite effect. The grafting method can organically combine GO with WPU through covalent and non-covalent bonds, and improve the compatibility and dispersion of GO in WPU [37,38]. Combining diisocyanate [39] or silane-functionalized [40] modified graphene oxide with WPU can improve the thermal, mechanical, and barrier properties of the coating [41,42]. Although the GO modification method can improve the mechanical properties and barrier properties of the coating, there still exist some problems such as complicated preparation process and high raw material cost. Therefore, we grafted GO directly onto polyurethane macromolecules in the process of in situ polymerization of monomers (Figure 1), and the intermolecular forces of polyurethane were improved to obtain Go-WPU coatings with high mechanical properties, high wear resistance, and strong solvent resistance. First, we prepared nanoscale GO by HUMMER method, and carried out microscopic observation and chemical characterization. Then, we prepared Go-WPU composite coating film with different GO concentration ratios, analyzed the micromorphology and chemical composition of GO-modified WPU, and explored the influence law of GO on the mechanical properties and solvent resistance of WPU. The small dose of GO can be organically bonded with WPU through covalent and non-covalent bonds, which improves the dispersion of GO in WPU. Meanwhile, the introduction of GO makes the molecular chains of linear WPU form a mesh structure, which improves the intermolecular bonding force and thus the degree of cross-linking. In addition, the comprehensive performance of GO-modified WPU is better than that of commercial coatings, which is suitable for all kinds of wood products and has a broad application prospect in the protection of wood products.

## 2. Materials and Methods

### 2.1. Materials

Isophorone diisocyanate (IPDI, 99%, Jining Hongming, Jining, China) was chosen as the hard segment for the WPU. Polypropylene glycol 2000 (PPG2000, 99%, Qingdao Yousuo, Qingdao, China) was used as the soft segment. 1,4-Butanediol (BDO, 99%, Shandong Xiya, Linyi, China) was used as the chain extender. Dimethylolpropionic acid (DMPA, 99%, Shanghai Macklin, Shanghai, China) was used as a chain extender and emulsifier. N-methyl-pyrrolidone (NMP, 99%, Shanghai Macklin, Shanghai, China) and acetone (99%, Shanghai Macklin, Shanghai, China) were used as organic solvents. Dibutyltin dilaurate (DBTDL, 98%, Tianjin Damao, Tianjin, China) was used as a catalyst. Triethylamine (TEA, 99%, Tianjin Kaitong, Tianjin, China) was used as a neutralizer for the WPU. Graphite powder (300 mesh, Qingdao Hengli, Qingdao, China) was used for the GO preparation. All chemical reagents were used directly without further purification.

### 2.2. Preparation of GO

First, graphite powders were placed and stirred in a beaker with sulfuric acid and sodium nitrate under an ice-water bath, and potassium permanganate was slowly added. Second, the temperature was increased to 35 °C and stirred for 2 h, and 115 mL of distilled water was added. Third, the temperature was raised to 95 ± 3 °C and maintained for 30 min, 10 mL of hydrogen peroxide solution (30 wt%) was added until no bubbles and golden yellow color appeared. Fourth, the supernatant was removed by allowing to stand and by stratifying, and the solution was washed several times with dilute hydrochloric acid (1 M) and deionized water to remove the metal ions and the acid, respectively. The resulting suspension (pH 7) was centrifuged at 8000 r/min, followed by the dialysis in deionized water for one week. Finally, the prepared GO was added to the deionized water and purified using an ultrasonic cell crusher to obtain a homogeneously dispersed GO gel suspension.

### 2.3. Preparation of GO-WPU Composite Emulsion

The preparation of GO-WPU composite emulsion was divided into three stages. The first stage was the pre-polymerization reaction, in which IPDI and GO were added to the four-necked flask, and maintained at 65 °C for 1 h, followed by the addition of PPG-2000 for 2 h, and 3 wt% of DBTDL was added dropwise. The second stage was the chain expansion reaction, in which BDO was mixed with 15 mL of acetone, stirred, and added dropwise to the reaction system, and maintained at 70–80 °C. After a complete reaction, DMPA (dissolved in NMP) containing hydrophilic groups was introduced. The third stage was the neutralization reaction, in which triethylamine was added to the reaction flask at 40 °C for 30 min. Finally, the obtained prepolymer was cooled to room temperature, the distilled water and the prepolymer were mixed with a high-speed disperser and evenly dispersed to obtain GO-WPU emulsion, and the composite emulsion was treated with ultrasound. A composite emulsion of 0.1 wt%, 0.4 wt%, 0.7 wt%, and 1 wt% GO-WPU was labeled as GO-WPU-0.1, GO-WPU-0.4, GO-WPU-0.7, and GO-WPU-1, respectively. The emulsion without GO was labeled WPU, which was used as a control group. The commercial WPU coating was labeled as CAP. Air bubbles generated during the mixing process were removed from all emulsions by vacuum treatment before.

### 2.4. Preparation of Coating

The emulsion (10 g) was placed in a polytetrafluoroethylene molding and dried at 60 °C for 1 h. The pure resin and the GO-modified composite film were labeled WPU and GO-WPU, respectively. Before applying the emulsions, two waterborne wood sealer primer emulsions were sprayed continuously onto the cork floor surface to form a thin layer of approximately 100 μm. First, one wood sealer primer was sprayed on the surface of virgin maple veneer, dried at 60 °C for 1 h, and then polished with 400# sandpaper. Next, another layer of waterborne woodwork primer was applied to the above surface, followed by the drying and polishing process described above. Finally, the emulsion used in this study was applied to the surface of the target cork flooring with a coating thickness of approximately 300 μm.

### 2.5. Structural Characterization and Performance Testing

The microscopic morphology of GO was observed using an atomic force microscope (AFM, FM-Nanoview1000, Suzhou FSM Precision Instruments Co., Ltd., Suzhou, China) and a field emission scanning electron microscope (FE-SEM, S4800, Hitachi, Ibaraki, Japan). The chemical composition of GO, graphene, and the composite coating film was investigated by employing Fourier transform infrared spectroscopy (FTIR; IRTracer-100, Shimadzu, Japan), UV-Vis-NIR Raman spectrometer (Raman; LabRAM HR800, HORIBA Jobin Yvon, Paris, France), and X-ray electron spectroscopy (XPS, ESCALAB 250Xi, Thermo Fisher Scientific, Waltham, MA, USA). The crystalline structures of the coating films were confirmed by adopting X-ray diffraction (XRD; D8 Advance, Bruker, Germany). Thermogravimetric analysis (TGA; SDT Q500, TA, New Castle, DE, USA) was used to test the thermal stability of the composite coating film.

The viscosity of the emulsion was tested by a digital display rotary viscometer (NDJ-5S, Lichen Instrument Technology Co., Ltd., Shaoxing, China). The deionized water was used to dilute the emulsion to a suitable concentration, and then the particle size analyzer (ZS-920, Shanghai Zimeng Technology Co., Ltd., Shanghai, China) was used to test the particle size of the emulsion. The tensile strength and elongation at break of the coating film were tested using the electronic universal testing machine (UTM2502, SUNS, Shenzhen, China) according to GB/T 13022-1991. The hardness test and the hardness test of the coating were conducted by a pendulum durometer (BGD 508, With Beijing’s Venture, Beijing, China) and a hand-cranking pencil durometer (QHQ, Huaguo Precision Instrument, Dongguan, China) according to GB/T 1730-1993 and GB/T 6739-2006, respectively. The abrasion durability of the coating was tested by a coating film abrasion tester (BGD 523, With Beijing’s Venture, Beijing, China) according to the GB/T1768-2006. The water absorption of the coating was measured concerning HG/T3344-2012. Cut the paint film into 3 cm × 3 cm, weigh it, and record it as m_1_, put it into 5% sodium hydroxide and ethanol respectively at 25 °C, soak it for 24 h, dry the surface water with filter paper, weigh it, and record it as m_2_. The formula of paint film dissolution rate is: $\frac{m_{2}-m_{1}}{m_{1}}\times 100\%$. The glossiness test of the coating was carried out via a three-angle gloss tester (GZ-II, World Expo Weiye, Beijing, China) according to GB/T 4896.6-2013. The coating adhesion of the coating was measured by a coating dagger (BGD 504/1, JONLN, Shanghai, China) according to GB/T 4893.4-2013. The contact angle of the coating film was measured by a video optical contact angle meter (OCA15EC, DATA PHYSICS, Filderstadt, Germany). All the results were repeated three times to obtain the average value.

## 3. Results

**Figure 2:**
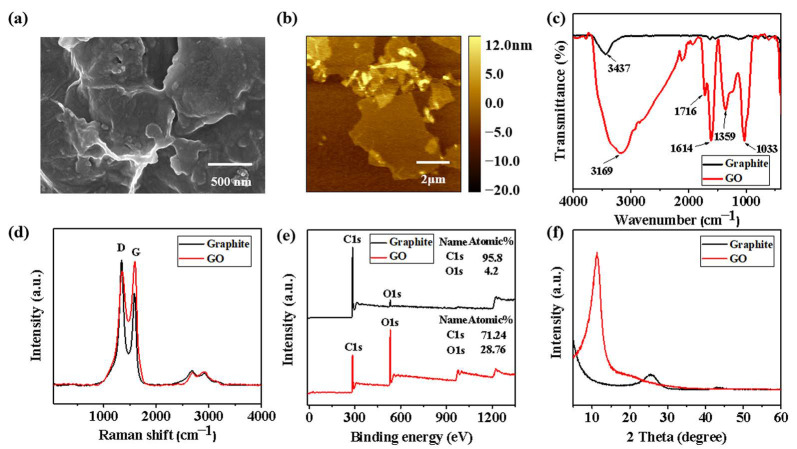
**Microscopic morphology and chemical characterization of GO.** (**a**) SEM morphology and (**b**) AFM morphology of GO; (**c**) FTIR spectroscopy, (**d**) Raman spectroscopy, (**e**) XPS spectra, and (**f**) XRD pattern of rGO and GO.

Figure 2a,b show the SEM and AFM images of the prepared GO, respectively. It can be clearly seen that the GO was a nanolayer sheet-like structure with some folds and stacks. Figure S1 clearly shows that the thickness of graphite was about 4 nm and the diameter varies from several hundred nanometers to several micrometers. Subsequently, the chemical structures of rGO and GO were evaluated and compared by FTIR, Raman, XPS and XRD spectroscopy, respectively. The peak at 1630 cm^−1^ was attributed to C=C stretching vibration (Figure 2c), which was also due to sp^2^ hybridization, and a small and narrow stretching vibration from the -OH peak was detected at 3437 cm^−1^, which might be that it is still slightly oxidized during the preparation process. GO showed a new series of FTIR absorption peaks compared to rGO, with a broader and stronger -OH stretching vibration peak in the range of 3000–3700 cm^−1^. The deformation vibration absorption peak corresponding to water molecules at 1614 cm^−1^ indicated that the GO was dried but water molecules are still presented, which was consistent with the incomplete drying of GO. The absorption peak at 1716 cm^−1^ was attributed to the telescopic vibrational peak of C=O on the GO carboxyl group; the absorption peak at 1033 cm^−1^ was attributed to the vibrational absorption peak of C-O-C. This indicated that at least three functional groups of -OH, -COOH and -C=O were presented in GO under the present experimental conditions [43]. The results of Raman spectrograms show that the characteristic peak near 1594 cm^−1^ is the “G peak” of graphite-like products, which represents the SP^2^ hybridized structure of carbon atoms; the characteristic peak near 1351 cm^−1^ corresponds to the “D peak”, which represents the SP^3^ hybridized structure of carbon atoms. The intensity ratio (ID/IG) of the D-band to the G-band is an indicator of the relative disorder structure. The ID/IG of graphite, GO, and rGO is 0.7 [44,45], 1.359, and 0.925, respectively. This intensity ratio indicates that defects, vacancies, and distortions are produced in GO during oxidation, which is mainly attributed to the appearance of oxygen-containing functional groups.

The XPS spectrum (Figure 2e) showed that the carbon-to-oxygen ratio of graphite was 30.25, while the carbon-to-oxygen ratio of GO was 2.5. It indicated that during the preparation of GO, a large number of oxygen-containing groups were generated and the carbon-to-oxygen ratio increased. Moreover, graphite showed a weak diffraction peak at 2θ = 25.47° with a layer spacing of 0.35 nm (Figure 2f). In contrast, GO exhibited a strong and sharp (001) peak at 2θ = 11.29° and the layer spacing was 0.78 nm. The layer spacing of GO was larger than that of rGO, and it could be inferred that GO had a larger layer spacing because of the insertion of oxygen-containing groups and GO generated a new crystal structure [46].

The FTIR, Raman, XPS, and XRD chemical characterization of GO showed that GO was rich in oxygen-containing functional groups, and its SEM and AFM microscopic morphology also demonstrated that GO is a two-dimensional sheet material, indicating that GO was successfully synthesized. In addition, the lamellar structure was dispersed in the polyurethane coating and had a positive effect on the chemical resistance of the coating.

**Figure 3:**
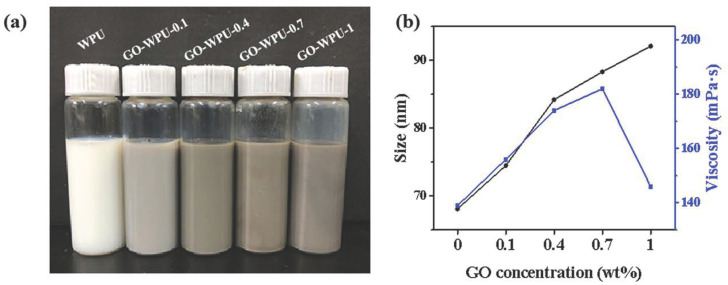
Effect of GO concentration on emulsions. (**a**) Appearance of emulsions after centrifugation; (**b**) particle size and viscosity of emulsions.

Figure 3a shows the appearance of the emulsions after centrifugation and settling. The WPU emulsions were creamy white and the color of the GO-WPU emulsions gradually increased with increasing GO concentration. The GO-WPU-1 emulsions produced some precipitation after centrifugation, so the color of the emulsions appeared slightly lighter than that of the GO-WPU-0.7 emulsions. The particle size and viscosity of WPU and GO-WPU emulsions were plotted against GO concentration in Figure 3b. The particle size of the WPU emulsion was 68.1 nm, and the particle size of the emulsion was positively correlated with GO concentration, gradually increasing to 92.1 nm of GO-WPU-1 emulsion. The viscosity of GO-WPU emulsion was also positively correlated with the increase of GO concentration and gradually increased to 196 mPa.s of GO-WPU-0.7 emulsion. The above results showed that GO grafting into the polyurethane molecular chain changed the molecular structure of the originally chain-like molecules into a net-like structure, which increased the molecular weight of polyurethane, thus increasing the particle size of GO-WPU emulsion and making the emulsion viscous. When the GO concentration reached 1 wt%, the viscosity of the emulsion decreased, probably due to the high polymerization of the emulsion and the precipitation of the emulsion, which reduced the solid concentration of the emulsion, which echoed the precipitation of the GO-WPU-1 emulsion in Figure 3a. The emulsion performance test showed that the reservoir stability of GO-WPU-1 emulsion was unsuitable for use and production. Therefore, we characterized and tested the performance of WPU, GO-WPU-0.1, GO-WPU-0.4, and GO-WPU-0.7 emulsions to investigate the effect of different GO concentrations on WPU coating performance.

**Figure 4:**
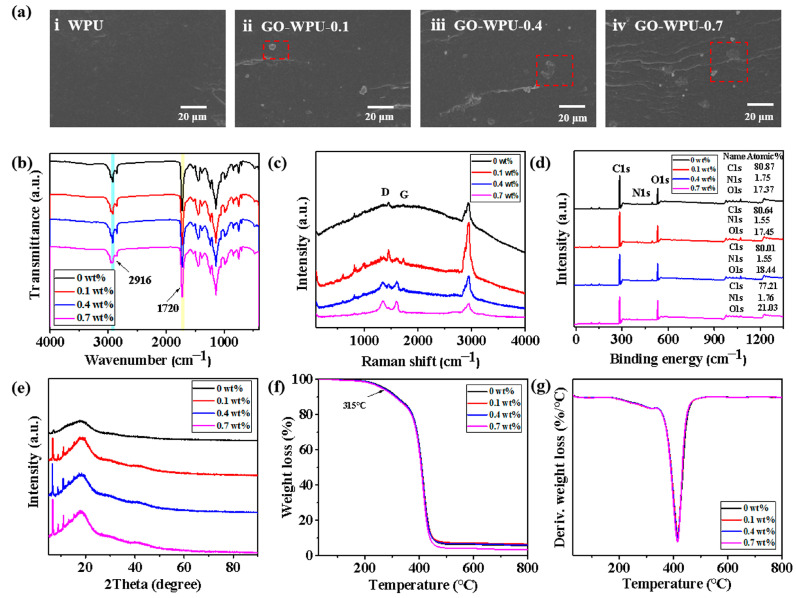
Effect of GO concentration on coating film performance: (**a**) SEM cross-sectional morphology, (**b**) FTIR spectra, (**c**) Raman spectra, (**d**) XPS diagrams, (**e**) XRD diagrams, (**f**) TG curves, and (**g**) DTG curves of the coating film.

The film sections were prepared by using the low-temperature liquid nitrogen brittle fracture method, and the microscopic morphology of the sections was observed by SEM, as shown in Figure 4a. GO were dispersed relatively and uniformly in WPU and without producing large agglomerates, but a little cloud-like rough defect appeared at the cross-sections of the GO-WPU coating films (in the red box). Meanwhile, the cross-sections became increasingly rough as the GO concentration increased. At the same time, the cross-section became rougher with the increasing GO concentration. This might be caused by the polar groups of GO leading to agglomeration and the appearance of defects [47]. Moreover, for the same reason, the glossiness of the WPU-GO coatings showed a gradual decrease with increasing GO concentration (Figure S2). The FTIR spectra of the coatings are presented in Figure 4b. It can be observed that there are only weak variations between GO-WPU and WPU due to the relatively low introduced concentration of GO, with a diminished peak only at the C=O (1710–1750 cm^−1^) of GO-WPU. The C=O peak at 1720 cm^−1^ is attributed to the ordered hydrogen bonding, reflecting the weakening of the hydrogen bonding (N–H···O=C) between the soft and hard segments or between the hard segments of WPU. The rich oxidation functional groups present on the GO surface were connected to the polyurethane segment by hydrogen bonding. In addition, all spectra showed no absorption peaks around 2200 cm^−1^, indicating the complete reaction of the free –NCO group in the system [48]. Considering that –OH on GO also formed carbamate group –NHCO with the resin –N=C=O group, the –NHCO group formed in GO-WPU is different from that of WPU. Based on the FTIR results, it can be inferred that GO exists in two ways: mixed with WPU through hydrogen bonding or embedded in the hard segment of WPU through in situ polymerization.

The Raman spectra of the coatings are shown in Figure 4c. Compared to pure WPU, the GO-WPU produced two new characteristic bands which came from the D-band (1350 cm^−1^) and G-band (1618 cm^−1^) of GO. The peaks of the D-band and G-band of GO-WPU gradually increased with the increase in GO concentration. In addition, the characteristic peak of C-H near 2916 cm^−1^ corresponded to the characteristic peak in the FTIR spectrum [49,50]. The XPS spectra showed a carbon-oxygen ratio of 4.66 for WPU, and 4.62, 4.33, and 3.67 for GO concentration of 0.1 wt%, 0.4 wt%, and 0.7 wt%, respectively (Figure 4d). The results indicated that the grafting of GO onto the polyurethane molecular chain allowed the introduction of numerous oxygen-containing groups into the polyurethane, which resulted in an increase in the carbon-oxygen ratio of GO-WPU. Compared with the XRD diffraction curves of the coatings (Figure 4e), WPU and GO-WPU had only one broad diffraction peak at around 17.8°. GO-WPU showed the characteristic peak of GO at about 10° corresponding to the XRD diffraction peak of GO. The morphologies of the diffraction peaks of WPU and GO-WPU were similar, and the intensity of the diffraction peaks gradually increased with the increase of GO concentration, which implied the increase of crystallinity. The result suggested that GO restricted the motion of chain segments enhanced the regularity of chain segments, and promoted the crystallization of WPU chain segments. The thermogravimetric (TG) curve of the coatings is shown in Figure 4f. The GO-WPU thermogravimetric curve were similar to WPU, with a 10% weight loss of around 315 °C. The TG curve mainly consisted of three degradation stages. The first stage presented a slight weight loss of about 5 wt% (T5%), which might be related to water evaporation. The second and third stages led to a weight loss of 10 ~ 80 wt% (T10 ~ T80%), which occurred successively with the decomposition of the hard and soft segments in the WPU chain [51]. WPU and GO-WPU generally had high thermal decomposition temperatures, which indicated that the introduction of GO would not significantly reduce the excellent thermal stability possessed by WPU itself.

The cross-sectional morphology of the coating films showed that GO was relatively uniformly dispersed in the WPU coating films and without producing large agglomerates. The results of chemical analysis and characterization also showed that the hydroxyl groups on the surface of GO were esterified with the isocyanate in the hard segment of polyurethane, which effectively improved the intermolecular force of polyurethane. In addition, a part of the oxygen-containing groups of GO also had hydrogen bonding interaction with polyurethane molecules, and this interaction also had a positive effect on the degree of cross-linking between polyurethane molecules. The results demonstrated that GO could produce good compatibility with WPU and that the two were tightly bonded through chemical/hydrogen bonding interactions.

**Figure 5:**
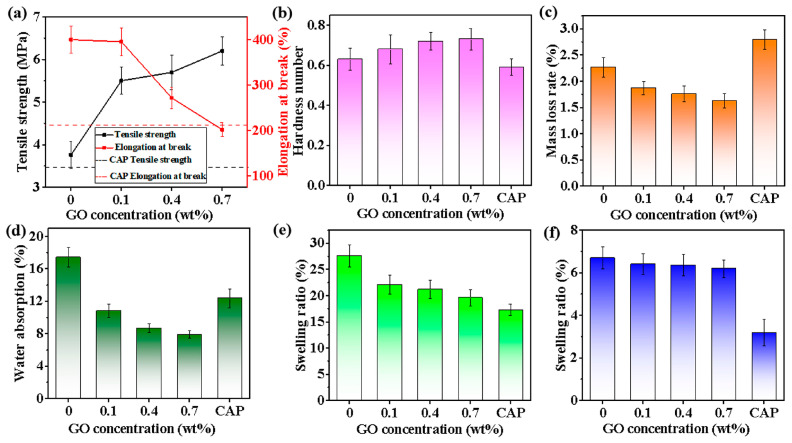
Physical performance tests of coating and coatings: (**a**) Tensile strength and elongation at break, (**b**) pendulum hardness, (**c**) abrasion durability, and (**d**) water absorption rate of coating film; swelling rate of (**e**) ethanol and (**f**) NaOH absorption by coating film.

The mechanical properties of the coating film have a critical impact on the reliability and durability of the resin in practical applications. As expected, the tensile strength of the GO-WPU coating film increased continuously and the elongation at break decreased with the gradual increase of GO concentration (Figure 5a). The peak value of tensile strength could be 6.1 MPa at 0.7 wt%, with a 64.89% improvement compared to WPU coating film, much higher than the control group CAP. However, its elongation at break decreased by 49.5%, still similar to the control CAP. This is mainly because GO is uniformly grafted on polyurethane molecules, which increased the molecular weight of the emulsion, and the excellent mechanical properties of GO and the hydrogen bonds formed by the abundant oxygen-containing functional groups on its surface enhanced the intermolecular interactions, thus improving the tensile strength. The introduction of the two-dimensional structure of GO enhanced the WPU intermolecular interactions and the increase in crystallinity made the deformation and slip of molecules inside the GO-WPU coated film smaller during stretching, attributed to the GO nanofillers hindering the mobility of the WPU matrix and eventually leading to a continuous decrease in elongation at break [52]. Compared with the pendulum hardness value of the WPU coating (0.63, Figure 5b), the pendulum hardness of the GO-WPU coating increased with the increase of GO concentration. The pendulum hardness reached a peak of 0.73 at 0.7 wt%, which was 15.87% higher than the WPU coating and 23.73% higher than the control CAP. The pencil hardness also showed the same increasing trend as the pendulum hardness (Table S1). GO increased the molecular weight of the resin molecules through chemical bonding and increased the interaction between resin molecules through hydrogen bonding, which in turn increased its resistance to external pressure.

The abrasion durability of the coating is also an important indicator in the practical application of the resin. The abrasion durability results showed that the mass loss rate of WPU coating was 2.27% (Figure 5c). The abrasion durability gradually increased with increasing GO concentration, and the mass loss rate of GO-WPU coating decreased to 1.87% at 0.1 wt% of GO concentration. The GO-WPU coating had the lowest mass loss rate of 1.63% at 0.7 wt%, much smaller than the mass loss rate of the control CAP (2.79%). The introduction of GO had a positive effect on the abrasion durability of the coating. The grafting of GO into the polyurethane molecular chain through chemical bonding significantly enhanced the interaction force between the molecules of the coating resin matrix. As the coating was subjected to mechanical wear, external forces mainly produced shear and tensile effects on the resin molecules, while the stronger mechanical properties of GO and the enhanced intermolecular forces of the resin improved the resistance of the coating to the shear and tensile forces produced by external mechanical wear [53]. The adhesion of various coatings is presented in Table S1. At GO concentration below 0.4 wt%, the adhesion of GO-WPU coating was not changed significantly compared with that of WPU coating, and its adhesion grade was 1. When the GO concentration reached 0.7 wt%, the adhesion of GO-WPU coating was slightly reduced and the adhesion grade became 2. This is probably due to the increased addition of GO and the enlarged particle size of the emulsion, which reduced the anchoring effect of the GO-WPU coating and showed a slight decrease in adhesion. In general, the introduction of GO enhanced the mechanical properties of the coating.

The water absorption of the WPU coating film was 17.41%. The water absorption rate of GO-WPU coating film decreased gradually with the increase in GO concentration. The water absorption was reduced to 7.89% when the GO concentration was 0.7 wt%, which was 54.7% and 36.2% lower than the WPU coating film and the control CAP, respectively (Figure 5d). The reason for the superior water resistance of GO-WPU coating film is that GO increased the molecular weight of resin and the interaction between resin molecules, which reduced the molecular gap and increased the denseness of the molecular chain in GO-WPU coating film. The two-dimensional structure of GO also made the movement path of water molecules into the interior of the coating film longer, so GO-WPU coating films exhibited excellent water resistance [54]. Figure S3 shows the contact angle of GO-WPU coating, and the contact angle increased gradually with the increase of GO concentration. At the microscopic level, the surface of GO-WPU coating became rougher with the increase of GO concentration, which made a liquid film of molecular action radius thickness exist at the contact between water molecules and the surface of GO-WPU coating. For a short period of time, the molecules of this thin layer were subjected to the adhesion of molecules inside the GO-WPU coating less than the cohesion of water molecules, resulting in a transient unwetting phenomenon that also retards the entry of water [55].

Figure 5e,f show the swelling rate of the coating film in ethanol and NaOH solutions. The swelling rate of the WPU coating film in ethanol solution was 27.6%. The swelling rate of GO-WPU coating film decreased to 19.6% at the GO concentration of 0.7 wt%, which was 28.9% lower and close to the swelling rate of the control CAP (17.3%). The swelling rate of WPU coating film in NaOH solution was 6.69%, and the swelling rate of GO-WPU coating film decreased to 6.19% at 0.7 wt% of GO concentration, which was 10.6% lower. Although there was a difference in the swelling rate (3.2%) compared to the control CAP group, it still showed a promising improvement. After the introduction of GO, the alcohol and alkali resistance of GO-WPU coating films also exhibited the same enhancement effect as water resistance, reflecting the general improvement of the solvent resistance of the coating films by the introduction of GO [56]. In addition, the alcohol and alkali resistance of the CAP film was better than that of the GO-WPU film, which could be attributed to the additives in CAP. We will also work on additives in our future work.

The performance test results show that the introduction of two-dimensional GO increased the molecular weight of resin molecules improved the strength of hydrogen bonds inside the coating, and had a positive impact on the improvement of various properties of the coating. The tensile strength, hardness, and abrasion resistance of GO-WPU coating film were significantly improved at 0.7% of GO addition, which indicated that GO and WPU maintain good compatibility and dispersion with strong intermolecular forces at this addition level. The results of water, alcohol, and alkali resistance also confirmed the contribution of GO to the solvent resistance of WPU coatings. These results showed that GO-WPU has promising application potential.

## 4. Conclusions

In this study, to improve the mechanical properties and solvent resistance of WPU, GO was prepared by the HUMMER method and grafted onto the molecular chain of WPU by chemical grafting, then the GO-WPU was prepared. The results showed that GO is grafted onto the molecular chain of WPU by chemical bonding and dispersed uniformly in the coating. When the GO concentration is 0.7 wt%, GO’s excellent mechanical properties and the abundant oxygen-containing functional groups on its surface enhance the internal hydrogen bonding, resulting in the GO-WPU coating presenting a tensile strength of up to 6.2 MPa, which is 64.89% higher than that of the pure WPU coating; a high pendulum hardness of 0.73, which is 15.87% higher than that of the pure WPU coating; a low mass loss of 1.63%, which is 28.19% lower than that of the pure WPU coating. The mechanical properties and durability of the coating are significantly improved. In addition, the introduction of the two-dimensional structure GO into the GO-WPU coating improves the barrier property of the coating and shows good solvent resistance. This study can provide a reference for the performance improvement of wood coatings.

## Data Availability

Not applicable.

## Supplementary Materials

The following supporting information can be downloaded at: https://www.mdpi.com/article/10.3390/polym15040882/s1. Figure S1: AFM cross section of GO. Figure S2: Glossiness of paint films. Figure S3: Contact angle of paint films. Table S1: Other performance tests of paint films.
