# Supplementary material for: Improvement of Mechanical Properties and Solvent Resistance of Polyurethane Coating by Chemical Grafting of Graphene Oxide
Source: Polymers (Basel). 2023 Feb 10;15(4):882. doi: 10.3390/polym15040882 (PMC9963133; doi:10.3390/polym15040882)
Supplement: Supplementary file 1 [file polymers-15-00882-s001.zip › polymers-2151874-supplementary.pdf]

polymers

Supplementary Material

Improvement of Mechanical Properties and Solvent Resistance of Polyurethane Coating by Chemical Grafting of Graphene Oxide

Guotao Liang [1,†], Fengbiao Yao [1,†], Yanran Qi [1,†], Ruizhi Gong [1,†], Rui Li [2], Baoxuan Liu [3], Yueying Zhao [4], Chenglong Lian [1,4,*], Luming Li [5,*], Xiaoying Dong [1,*] and Yongfeng Li [1,*]

[1] State Forestry and Grassland Administration Key Laboratory of Silviculture in Down-Stream Areas of the Yellow River, College of Forestry, Shandong Agricultural University, Tai'an 271018, China; gtaoliang@163.com (G.L.); fengbiaoyao@163.com (F.Y.); qyran1994@163.com (Y.Q.); ruizhigong789@163.com (R.G.)
[2] School of Automation, Chongqing University of Posts and Telecommunications, Chongqing 400065, China; lirui@cqupt.edu.cn
[3] Shandong Laucork Development Co., Ltd., Jining 272100, China; liubaoxuan123@126.com.
[4] Postdoctoral Innovation Practice Base, Shandong Xiaguang Group Co., Ltd., Jining 277600, China; xgsyyf@126.com (Y.Z.)
[5] College of Chemistry and Materials Engineering, Zhejiang A&F University, Hangzhou 311300, China
* Correspondence: lyjj917@sdau.edu.cn (C.L.); llm@zafu.edu.cn (L.L.); dxiaoying1982@163.com (X.D.); lyf288@hotmail.com (Y.L.), Tel.: +86 538 8240610 (Y.L.)
† These authors contributed equally to this work.

Citation: Liang, G.; Yao, F.; Qi, Y.; Gong, R.; Li, R.; Liu, B.; Zhao, Y.; Lian, C.; Li, L.; Dong, X.; Li, Y. Improvement of Mechanical Properties and Solvent Resistance of Polyurethane Coating by Chemical Grafting of Graphene Oxide. *Polymers* **2023**, *15*, 882. https://doi.org/10.3390/polym15040882

Academic Editor(s): Sándor Kéki

Received: 25 December 2022
Revised: 19 January 2023
Accepted: 7 February 2023
Published: date

Copyright: © 2023 by the authors. Licensee MDPI, Basel, Switzerland. This article is an open access article distributed under the terms and conditions of the Creative Commons Attribution (CC BY) license (https://creativecommons.org/licenses/by/4.0/).

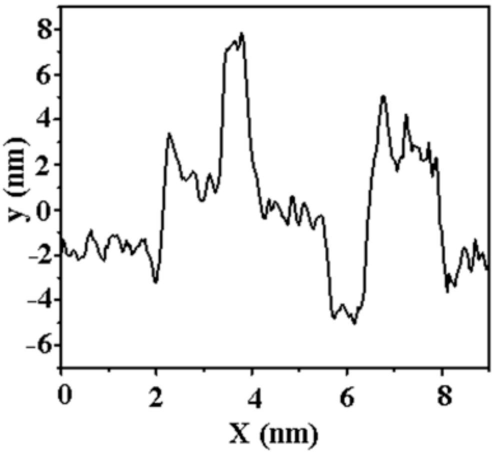

Figure S1. AFM cross section of GO.

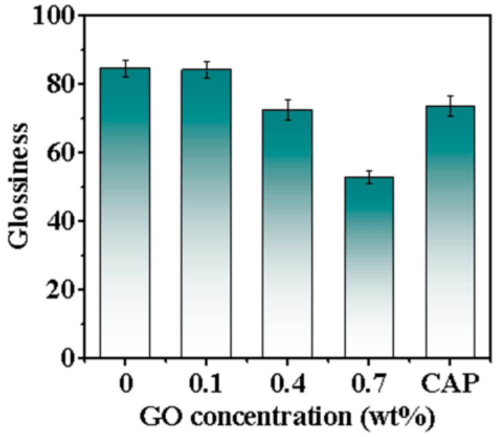

Figure S2. Glossiness of paint films.

Table S1. Other performance tests of paint films.

Sample	Surface Dring Times(min)	Hard Dring Times(min)	Penscil Hard-ness	Adhesion
WPU	29.6	55	2B	1
GO-WPU-0.1	29.5	55.3	B	1
GO-WPU-0.4	29.9	58.7	B	1
GO-WPU-0.7	30.2	61.6	B	2
CAP	33.9	72.6	2B	1

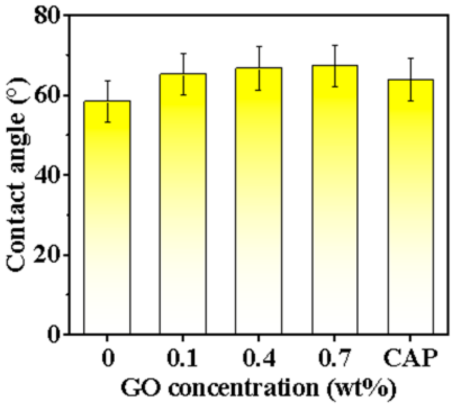

Figure S3. Contact angle of paint films.
